# Psychometric properties of the multidimensional assessment of interoceptive awareness (MAIA) in a Chilean population

**DOI:** 10.3389/fpsyg.2015.00120

**Published:** 2015-02-11

**Authors:** Camila Valenzuela-Moguillansky, Alejandro Reyes-Reyes

**Affiliations:** ^1^Instituto de Sistemas Complejos de ValparaísoValparaíso, Chile; ^2^Escuela de Psicología, Universidad Santo TomásConcepción, Chile

**Keywords:** interoceptive awareness, body awareness, multidimensional assessment of interoceptive awareness, psychometrics properties, mind–body

## Abstract

The multidimensional assessment of interoceptive awareness (MAIA) is an instrument designed to assess interoceptive awareness. The aim of this study was to adapt the original MAIA scale to Spanish and to analyze its psychometric properties in a Chilean population. The MAIA was administered to 470 adults, aged 18–70 years, 76.6% women and 23.4% men, residents of the provinces of Valparaíso and Concepción, Chile. Exploratory factor analysis reduced the scale from 32 to 30 items. Confirmatory factor analysis supports a structure of eight interrelated factors (Noticing, Not-Distracting, Not-Worrying, Attention Regulation, Emotional Awareness, Self- Regulation, Body Listening, and Trusting), similar to the original scale (χ^2^_(371)_ = 659.78, *p* = 0.0001; CFI = 0.92, TLI = 0.91, RMSEA = 0.056 and SRMR = 0.059). The Spanish version showed appropriate indicators of construct validity and reliability, with a Cronbach’s α of 0.90 for the total scale, and values between 0.40 and 0.86 for the different subscales. Similar to previous studies, low reliability was observed in two of the eight scales (Not-Distracting and Not-Worrying), thus further revision of these subscales is suggested. The Spanish version of MAIA proved to be a valid and reliable tool to investigate interoceptive awareness in the Chilean population.

## INTRODUCTION

This article presents the adaptation into the Spanish language of the multidimensional assessment of interoceptive awareness (MAIA) self-report instrument developed by Mehling et al. (2012), and the evaluation of its psychometric properties in the Chilean population.

Interoceptive awareness relates to the conscious perception of our internal state. Originally introduced by Sherrington (1906), the term interoception has been linked to visceral sensitivity, meaning the ability to detect the signals coming from our “internal milieu.” Recently, this term has been redefined as the sense of the physiological condition of the body and not only the viscera (Craig, 2002). This redefinition expands the notion of interoception, placing it as the afferent pathway of the autonomic nervous system. Under this view, afferent signals from the various tissues of the body, which contribute to the regulation of physiological parameters, constitute “a basis for the subjective evaluation of one’s condition,” in other words, the basis for *interoceptive awareness*. The link between interoception and interoceptive awareness opens the door to the potential mechanisms underlying the relationship between organic function of our body and our mental and emotional experience.

The meaning of “interoceptive awareness,” however, varies depending on the discipline and on the method used to evaluate it. For example, a method widely used to assess interoceptive awareness is cardiac monitoring. This method measures the person’s ability to detect his or her own heartbeat. In such a case, the internal state refers to the heartbeat signal, and interoceptive awareness is defined as the ability to count one’s own heartbeats. However, if we evaluate interoceptive awareness using the method of stimulation of the gastrointestinal tract, the internal state refers to the induced stimulation on the gastrointestinal tract and interoceptive awareness is defined as the ability to detect gastrointestinal signals. Typically, high interoceptive awareness assessed using such methods is related to maladaptive personality traits associated with states of anxiety and emotional liability (Schandry, 1981; Ehlers and Breuer, 1992). It is not clear whether such methods assess what Craig (2002) refers to with “subjective evaluation of one’s condition, that is, how you feel.” This description seems closer to a more global awareness of our internal state, such as being able to identify whether we feel at ease, or distressed. This kind of interoceptive awareness is heightened through practices, such as Yoga and Mindfulness Meditation, which develop a particular kind of attention toward the body and toward the person’s internal state, characterized by a receptive attitude (i.e., Ditto et al., 2006; Kabat-Zinn, 2008). While interoceptive awareness assessed by the methods measuring cardiac or gastrointestinal awareness focuses on perturbed physical states, awareness developed through these practices has a beneficial impact in a person’s physical and mental health (i.e., Chow and Tsang, 2007; Davis et al., 2007; Rosenzweig et al., 2007; Morone et al., 2008). These differences highlight that different ways of attending to the body, some adaptive while others not, might be grouped under the same concept.

The scale adapted in the present study aims to contribute in distinguishing these different modes of interoceptive awareness to serve as a tool for experimental interoception research, and for assessment of mind–body therapies.

Multidimensional assessment of interoceptive awareness was developed through a systematic mixed-methods process involving reviewing the current literature, specifying a multidimensional conceptual framework, evaluating prior instruments, developing items, and analyzing focus group responses to scale items by instructors and patients of body awareness-enhancing therapies (Mehling et al., 2012). It was refined by cognitive interviews, and items were field-tested in students and instructors of mind–body approaches. Field test data were submitted to an iterative process using multiple validation methods, including exploratory cluster and confirmatory factor analyses, comparison between known groups, and correlations with established measures of related constructs (Mehling et al., 2012). The resulting 32-item multidimensional instrument is composed of eight subscales: (1) Noticing: the awareness of uncomfortable, comfortable, and neutral body sensations; (2) Not-Distracting: the tendency to ignore or distract oneself from sensations of pain or discomfort; (3) Not-Worrying: emotional distress or worry with sensations of pain or discomfort; (4) Attention Regulation: the ability to sustain and control attention to body sensation; (5) Emotional Awareness: the awareness of the connection between body sensations and emotional states; (6) Self-Regulation: the ability to regulate psychological distress by attention to body sensations; (7) Body Listening: actively listening to the body for insight and (8) Trusting: experiencing one’s body as safe and trustworthy.

Multidimensional assessment of interoceptive awareness’s convergent and divergent validity was tested using different published measures of constructs related to body awareness. Aspects of mindful attention and body awareness were assessed with the Five Factor Mindfulness Questionnaire (FFMQ), the Private Body Consciousness Subscale (PBCS) of the Body Consciousness Questionnaire and the Body Responsiveness Questionnaire (BRQ). Aspects of anxiety as a state or trait, or as distress in response to bodily symptoms or pain, were assessed with the physical concern subscale (ASI-PC) of the Anxiety Sensitivity Index (ASI), the Pain Catastrophizing Scale (PCS) and the State-Trait Anxiety Inventory (STAI), which assessed convergent validity of the MAIA Not-Worrying subscale.

Multidimensional assessment of interoceptive awareness has been translated into nine languages. To our knowledge, the only adaptation to date that has published the assessment of its psychometric properties is the German version. Bornemann et al. (2014) analyzed whether the factor structure of the English MAIA would replicate in the German version. The exploratory factor analysis (EFA) showed that the German version has eight factors that group items in the same manner as the English version, with the exception of item 19 that loaded Emotional Awareness and Body Listening equally.

This article presents the translation and adaptation procedure of the MAIA tool to a Spanish version, and the evaluation of its psychometric properties applied to a Chilean population.

## METHODS

### PARTICIPANTS

The sample consisted of 470 participants, aged between 18 and 70 years (*M* = 30.52, *SD* = 10.60), from the provinces of Valparaíso and Concepción, Chile. 76.6% were female and 23.4% male with no statistically significant differences in age (*t*_(207.479)_ = 0.567, *p* = 0.572). The sample included undergraduate students (*n* = 205; 43.6%), graduate students (*n* = 98; 20.9%), university professionals from different areas (*n* = 142; 30.2%) and people with secondary or lower level of education (*n* = 25; 5.3%).

### INSTRUMENT

The MAIA is a self-administered instrument developed by Mehling et al. (2012) to measure eight dimensions of interoceptive body awareness. It has a total of 32 items tested on a Likert scale, with six levels of ordinal response coded from 0 (never) to 5 (always), generating a total direct score on a scale that ranges from 0 to 160 points. The number of items and reliability established by Cronbach’s alpha (α), vary among the subscales: noticing (four items, α = 0.69), Not-Distracting (three items, α = 0.66), Not-Worrying (three items, α = 0.67), Attention Regulation (seven items, α =0.87), Emotional Awareness (five items, α = 0.82), Self-Regulation (four items, α = 0.83), Body Listening (three items, α = 0.82) and Trusting (three items, α = 0.79). The Spanish version of the scale preserved the extension, format and dimensional structure of the original version.

### PROCEDURE

The Institutional Bioethics Committee of the University of Valparaíso (Chile) approved the study. Three stages were conducted for the translation and adaptation of the questionnaire: translation, cognitive interviews and survey.

#### Spanish translation

The translation was based on the original English version of MAIA. Before carrying out the translation, agreement was obtained from the first author of the scale [Wolf Mehling (W.M.)]. A forward–backward translation was performed comprising the following steps:

• Three independent forward translations were made: two by bilingual Spanish native translators who didn’t know the construct and one by a bilingual Spanish native person who was familiar with the construct.
• The three versions were compared and, after consensus between the two translators and the project manager, a single document was drafted.
• An English native bilingual translator, who was not familiar with the construct, performed the back-translation into English.
• Divergences between the back-translation and the original English version were identified and discussed with the first author of the original scale. For the items where cross-language agreement could not be reached, Spanish sentences were reworded.

#### Cognitive interviews

The cognitive interviews sample included thirteen people aged 21 to 72 (*M* = 42.8; *SD* = 15.6), with education level from high school to graduate school. Five persons were “body awareness-experienced.” Two persons had chronic pain. The sample was primarily female (*n* = 10).

Interviewees were asked to complete the MAIA and note next to each item whether they had any doubts or comments. On completing the survey, they filled a Participants Information Form and a cognitive interview was conducted. One half of participants were asked in-depth questions for all items while the other half were asked in-depth questions where they had noted concerns, or that had been identified as potentially conflicting by our research team. Interviews began with “Did this item make sense to you?” followed by “can you elaborate?” For the items identified as potentially conflicting, specific questions were elaborated. Results from the cognitive interviews were discussed with the first author of the scale, and changes were made when considered appropriate.

#### Survey

The scale was self-administered using a web platform with the exception of 90 undergraduate students who completed a paper survey. In both modalities (web-survey and paper), participants were explained the purpose of the study, were informed that they would not be compensated for their participation, that they were free to respond and that by agreeing to answer the scale they were giving their informed consent to participate in the study. In the web version this information was presented before the scale. In addition, it was explained that the research manager could be reached by email to respond to any questions concerning the study.

A participants information form was used to collect the demographic characteristics of participants (age, gender, educational level, presence of chronic pain, treatment, medication, and level of practice in five different body–mind techniques). This was followed by the Spanish version of the MAIA, after which there were two additional questions assessing whether participants had any problems with any of the scale items.

A pre-test with 12 subjects was conducted for the web-survey to verify comprehensiveness, ease of use of the web interface, and that data were correctly recorded, stored, and able to be exported. The mean duration of the survey was about 9 min, which was judged acceptable. The web platform used was Surveygizmo.

### DATA ANALYSIS

Missing values were imputed using the Markov Chain Monte Carlo (MCMC) method. From the total of 470 responses per item, there were 1–7 missing values in 28 items.

To evaluate the factorial structure of the scale, a cross-validation procedure was implemented where the total sample (*n* = 470) was randomly divided in two subsamples: a training sample (*n* = 220, 46.8%) and a validation sample (*n* = 250, 53.2%). The training sample was used to carry out an EFA to identify the factor structure of the MAIA. Estimation of the factors was performed by factoring the Pearson correlation matrix by the maximum likelihood (ML) method with an oblique Promax rotation. Parallel analysis (PA), the goodness of fit index (GFI) and the root mean square error of approximation (RMSEA) were used to select the factors. The validation sample was used to perform confirmatory factor analysis (CFA), testing the factorial structure obtained with the EFA.

Factor loadings with a minimum value in the range of ±0.30 were considered as building criteria for the EFA model (Hair et al., 2010). In the CFA, a good fit for a model was considered when the chi-square statistic (χ^2^) was not significant, the root mean square error of approximation (RMSEA) <0.08 and comparative fit index (CFI), GFI index and non-normed fit index (NNFI or TLI) > 0.95 (Hu and Bentler, 1999; Kline, 2011; West et al., 2012).

Cronbach’s alpha coefficient was used to establish the reliability of the scale and subscales. To examine associations between items and relationship between subscales, the Pearson correlation matrix was used.

Statistical analyses were performed using IBM SPSS Statistics 20 (IBM Corp.), IBM SPSS AMOS 18 (IBM Corp.) and FACTOR 9.30 (Lorenzo-Seva and Ferrando, 2006) programs.

## RESULTS

### TRANSLATION AND COGNITIVE INTERVIEWS

From the cognitive debriefing interviews we identified four main conflicting issues. These were discussed with the first author of the original scale (W.M.)

• Twelve out of thirty two items are formulated using “*Puedo*…” (*I can*…). This formulation appeared ambiguous to several interviewees since they didn’t know whether they should respond what they potentially could do, or what they actually do. This remark, more than a translation issue, was intentional in the design of the original scale. After discussion with W.M., we decided to leave the original formulation.
• The reverse item 5 was formulated using negation, which confused several interviewees. Mehling et al. (2013) also reported problems with item 5 and suggested: “…this item may have to be dropped or reworded in any future studies.” Consulting with W.M., we reformulated item 5, removing the negation: instead of *“I do not notice (I ignore) physical tension or discomfort until they become more severe,”* the Spanish version was “*Noto la tensión física o el malestar solamente cuando se vuelve muy severo”* [*I notice physical tension or discomfort only when they become very severe*].
• The reverse item 6 in the original English version (*I distract myself from sensations of discomfort*) was formulated using an affirmation. However, in Spanish, the formulation “*me distraigo*” was considered an active voluntary attitude that differed from the connotation in English. We translated this item using a negation: “*No me doy cuenta de las sensaciones de malestar*” [*I don’t notice sensations of discomfort*]. Some interviewees expressed confusion regarding this formulation. However, the negation was maintained following discussion with W.M.
• Some participants described certain items as lacking in context to situate an affirmation. For instance, some interviewees didn’t understand item 15 (*I can refocus my attention from thinking to sensing my body)*, or found it awkward, arguing that it depends on the context whether they would do that. Following review with W.M, we considered that this was a general issue of the MAIA depending on the participant’s familiarity with mind–body practices. Thus, we decided not to change the item.
• Although generally participants did not have problems with item 7 (*When I feel pain or discomfort, I try to power through it*), through the interviews we realized that the term *“sobrepasar*” used to translate “*to power through it”* was ambiguous. When asked what do they do to “*sobrepasar*” there was a variety of responses, and each participant attributed a different meaning to the word. After discussing with the first author of the original scale, we changed this item to: *“Cuando siento dolor o malestar intento ignorarlo y continuar con lo que estaba haciendo” [When I feel pain or discomfort, I try to ignore it and to carry on with what I was doing*].

### SURVEY

The assessment of assumptions necessary for the use of factor analysis showed a Kaiser–Meyer–Olkin (KMO) sampling adequacy of 0.884, and a significant Bartlett test of sphericity (χ^2^ = 3416.8; *p* < 0.001). This supports factor analysis as an appropriate model for analyzing the data. Since the items of the scale had an ordinal polyatomic response, assumption of a multivariate normal distribution is not met. Assessment of skewness and kurtosis showed that most values were in the range -1 and 1 (see **Table 1**). There were seven items that exceeded this criterion, but remained in the range -1.5–1.5: only Item 2 was outside this range. This allows inferring an approximation of each item to a Normal distribution (Lloret-Segura et al., 2014). Such statistics, coupled with the property that each item has six response levels, enables the use of the ML method to estimate the model parameters. This method has demonstrated robustness when the assumption of multivariate normality fails, and when there is an approximately normal univariate distribution (Forero et al., 2009).

**Table 1:** Univariate descriptives statistics for the items (*n* = 220).

Item	Mean	Confidence interval (95%)		Variance	Skewness	Kurtosis
Item 1	3.841	3.62	4.06	1.652	-1.146	0.853
Item 2	4.182	3.99	4.38	1.267	-1.669	2.762
Item 3	3.336	3.09	3.58	2.032	-0.674	-0.310
Item 4	3.782	3.54	4.02	1.971	-1.112	0.381
Item 5	2.400	2.15	2.65	2.104	0.061	-0.925
Item 6	3.277	2.96	3.59	3.282	-0.658	-1.022
Item 7	2.205	1.94	2.47	2.399	0.400	-0.806
Item 8	2.809	2.54	3.08	2.445	-0.318	-0.913
Item 9	2.136	1.87	2.40	2.336	0.252	-0.927
Item 10	2.045	1.78	2.31	2.352	0.257	-1.024
Item 11	2.850	2.59	3.11	2.328	-0.239	-0.936
Item 12	3.232	2.98	3.48	2.105	-0.544	-0.624
Item 13	3.077	2.81	3.35	2.444	-0.552	-0.760
Item 14	3.245	3.00	3.49	2.076	-0.628	-0.483
Item 15	3.345	3.12	3.57	1.653	-0.498	-0.269
Item 16	2.777	2.54	3.01	1.855	-0.068	-0.676
Item 17	3.127	2.89	3.36	1.820	-0.512	-0.336
Item 18	3.627	3.39	3.87	1.907	-0.974	0.199
Item 19	3.827	3.60	4.05	1.716	-1.238	0.970
Item 20	4.027	3.82	4.23	1.399	-1.294	1.053
Item 21	3.991	3.77	4.22	1.682	-1.390	1.278
Item 22	4.086	3.89	4.28	1.306	-1.308	1.218
Item 23	2.764	2.51	3.02	2.153	-0.305	-0.689
Item 24	2.655	2.41	2.90	2.008	-0.172	-0.730
Item 25	3.323	3.07	3.58	2.164	-0.690	-0.375
Item 26	2.841	2.58	3.10	2.252	-0.310	-0.912
Item 27	2.886	2.62	3.15	2.319	-0.304	-0.854
Item 28	2.355	2.09	2.62	2.392	0.074	-0.993
Item 29	2.400	2.14	2.66	2.240	-0.076	-0.942
Item 30	3.150	2.89	3.41	2.300	-0.405	-0.811
Item 31	3.200	2.94	3.46	2.269	-0.496	-0.701
Item 32	3.645	3.42	3.88	1.774	-0.987	0.318

Using the training sample (*n* = 220) successive factorial solutions were obtained using ordinary least squares (OLS) and ML method combined with the oblique rotations Direct Oblimin, Promin, and Promax. Factor solutions with six, seven, and eight factors were analyzed.

Results of the six-factor solutions generate a single factor for items 5, 6, 7, 8, and 9, but showed several factor loadings lower than 0.30. Items from 19 to 28 formed a single factor with factor loadings higher than 0.60. Solutions based on seven factors tended to differentiate two factors amongst items 5, 6, 7, 8, and 9. Items from 19 to 28 remained as a single factor, with factor loadings higher than 0.60 Among solutions based on eight factors, a model was found with loadings greater than or equal to 0.30, where seven of the eight factors comprised three or more items. The eight factors model achieved the greatest quality and was used to perform the CFA. As an analytic strategy, we used the ML method with normalized Promax rotation calculated with FACTOR 9.30. The factor structure matrix is shown in **Table 2**.

**Table 2:** Items and Exploratory Factor Analysis (EFA) loadings Spanish version of MAIA.

		Factors								Original scale	Spanish version
Items		1	2	3	4	5	6	7	8
**Noticing**
1	Cuando estoy tenso(a) noto dónde se ubica la tensión en mi cuerpo. *When I am tense I notice where the tension is located in my body. *	0.22	0.24	**0.73**	-0.04	0.26	-0.02	0.32	0.42	N	N
2	Me doy cuenta cuando me siento incómodo(a) en mi cuerpo. *I notice when I am uncomfortable in my body. *	0.24	0.10	**0.64**	0.02	0.41	0.14	0.45	0.41	N	N
3	Cuando estoy cómodo(a) lo noto en partes específicas de mi cuerpo. *I notice where in my body I am comfortable.*	0.32	0.21	**0.44**	-0.14	0.44	-0.10	0.37	0.37	N	N
4	Noto cambios en mi respiración. tales como cuando se hace más lenta o más rápida. *I notice changes in my breathing. such as whether it slows down or speeds up.*	0.17	0.20	0.34	-0.19	0.29	-0.09	**0.46**	0.40	N	_
**Not-Distracting**
5	Noto la tensión física o el malestar solamente cuando se vuelve muy severo. *I do not notice (I ignore) physical tension or discomfort until they become more severe.*	0.11	0.05	0.05	**0.53**	0.06	-0.01	0.00	0.08	ND	ND
6	No me doy cuenta de las sensaciones de malestar. *I distract myself from sensations of discomfort*	0.14	0.08	0.06	**0.40**	-0.04	-0.01	-0.00	0.08	ND	ND
7	Cuando siento dolor o malestar intento ignorarlo y continuar con lo que estaba haciendo. *When I feel pain or discomfort. I try to power through it.*	0.19	0.15	-0.08	**0.47**	0.15	-0.10	-0.02	0.10	ND	ND
**Not-Worrying**
8	Cuando siento dolor físico me enojo. *When I feel physical pain. I become upset.*	0.19	0.14	-0.16	**0.29**	0.05	0.17	-0.16	0.04	NW	_
9	Si siento alg\'{u}n malestar me empieza a preocupar que algo no ande bien. *I start to worry that something is wrong if I feel any discomfort.*	0.01	-0.04	-0.01	0.05	-0.08	**0.27**	-0.15	-0.08	NW	NW
10	Puedo sentir alguna sensación física desagradable sin preocuparme por ella. *I can notice an unpleasant body sensation without worrying about it.*	-0.04	0.01	0.07	-0.19	0.07	**0.46**	-0.08	0.14	NW	NW
**Attention Regulation**
11	Puedo prestar atención a mi respiración sin ser distraído(a) por lo que pasa a mi alrededor. *I can pay attention to my breath without being distracted by things happening around me.*	0.38	0.45	0.27	-0.09	0.54	0.11	0.39	**0.70**	A	A
12	Puedo tener conciencia de mis sensaciones corporales internas aun cuando hay muchas cosas sucediendo a mi alrededor. *I can maintain awareness of my inner bodily sensations even when there is a lot going on around me.*	0.31	0.27	0.37	-0.02	0.57	0.26	0.38	**0.69**	A	A
13	Cuando estoy conversando con alguien puedo prestarle atención a mi postura. *When I am in conversation with someone. I can pay attention to my posture.*	0.32	0.29	0.34	0.06	0.32	-0.12	0.27	**0.62**	A	A
14	Puedo volver a concentrarme en mi cuerpo si estoy distraído(a). *I can return awareness to my body if I am distracted.*	0.42	0.44	0.41	-0.00	0.45	-0.04	0.36	**0.78**	A	A
15	Puedo re-dirigir mi atención desde mis pensamientos a mis sensaciones corporales. *I can refocus my attention from thinking to sensing my body.*	0.41	0.41	0.33	0.07	0.45	0.02	0.31	**0.65**	A	A
16	Puedo prestar atención a todo mi cuerpo incluso cuando una parte de mi siente dolor o malestar. *I can maintain awareness of my whole body even when a part of me is in pain or discomfort.*	0.51	0.48	0.48	-0.29	0.37	-0.29	0.27	**0.72**	A	A
17	Soy capaz de concentrarme conscientemente en mi cuerpo de manera global. *I am able to consciously focus on my body as a whole.*	0.50	0.52	0.33	-0.02	0.48	-0.08	0.34	**0.77**	A	A
**Emotional Awareness**
18	Noto cómo mi cuerpo cambia cuando estoy enojado(a). *I notice how my body changes when I am angry.*	0.21	0.26	0.50	-0.29	0.53	-0.12	**0.60**	0.35	E	E
19	Cuando algo anda mal en mi vida puedo sentirlo en mi cuerpo. *When something is wrong in my life I can feel it in my body.*	0.19	0.22	0.52	-0.22	0.40	-0.27	**0.53**	0.30	E	E
20	Noto que mi cuerpo se siente diferente despu\'{e}s de una experiencia apacible. *I notice that my body feels different after a peaceful experience.*	0.30	0.30	0.51	-0.07	0.35	0.02	**0.75**	0.45	E	E
21	Noto que puedo respirar libre y fácilmente cuando me siento cómodo(a). *I notice that my breathing becomes free and easy when I feel comfortable.*	0.34	0.38	0.31	-0.04	0.33	-0.11	**0.78**	0.39	E	E
22	Noto cómo mi cuerpo cambia cuando me siento contento(a)/feliz. *I notice how my body changes when I feel happy / joyful.*	0.30	0.31	0.31	-0.13	0.40	-0.11	**0.82**	0.36	E	E
**Self-Regulation**
23	Cuando me siento sobrepasado(a) puedo encontrar un lugar tranquilo dentro de mi. *When I feel overwhelmed I can find a calm place inside.*	0.55	**0.62**	0.33	-0.11	0.44	-0.10	0.30	0.51	S	S
24	Cuando dirijo la atención hacia mi cuerpo siento calma. *When I bring awareness to my body I feel a sense of calm.*	0.60	**0.78**	0.34	-0.17	0.45	-0.33	0.25	0.50	S	S
25	Puedo utilizar mi respiración para reducir la tensión. *I can use my breath to reduce tension.*	0.47	**0.82**	0.20	0.08	0.45	-0.02	0.40	0.54	S	S
26	Cuando estoy atrapado(a) en mis pensamientos puedo calmar mi mente concentrándome en mi cuerpo/respiración. *When I am caught up in thoughts. I can calm my mind by focusing on my body/breathing.*	0.46	**0.84**	0.20	0.03	0.54	-0.02	0.34	0.48	S	S
**Body Listening**
27	Estoy a la escucha de la información que envía mi cuerpo sobre mi estado emocional. *I listen for information from my body about my emotional state.*	0.46	0.62	0.23	0.08	**0.75**	-0.13	0.40	0.62	B	B
28	Cuando estoy alterado(a). me tomo el tiempo para explorar cómo se siente mi cuerpo. *When I am upset. I take time to explore how my body feels.*	0.48	0.54	0.30	-0.05	**0.80**	-0.06	0.34	0.53	B	B
29	Escucho a mi cuerpo para saber qué hacer. *I listen to my body to inform me about what to do.*	0.57	0.58	0.41	-0.10	**0.71**	-0.19	0.27	0.63	B	B
**Trusting**
30	En mi cuerpo. estoy en casa. *I am at home in my body.*	**0.81**	0.50	0.19	0.05	0.46	-0.08	0.32	0.53	T	T
31	Siento que mi cuerpo es un lugar seguro. *I feel my body is a safe place.*	**0.96**	0.56	0.31	0.09	0.41	-0.10	0.25	0.51	T	T
32	Confío en mis sensaciones corporales. *I trust my body sensations.*	**0.68**	0.46	0.20	0.14	0.40	-0.07	0.31	0.42	T	T
	Proportion of variance	31.2	8.1	5.4	5.3	4.4	3.8	3.5	3.3		

*N, Noticing; ND, Not-Distracting; NW, Not-Worrying; A, Attention Regulation; E, Emotional Awareness; S, Self-Regulation; B, Body Listening; T, Trusting. Method for factor extraction: ML, Maximum Likelihood; Method the rotation: Normalized Promax. Items 5, 6, 7, 8, and 9 of the Spanish version are reverse. Bold values are factor loading maximo.*

The commonalities reproduced by the rotated factor solution ranged between.36 and.93, where the eight extracted factors explained 67.2% of the total variance. The factorial structure had low factor loading in items 8 (0.29) and 9 (0.27), which does not allow specifying the Not-Worrying subscale. The remaining factor loadings were above.40, considered significant for a sample size of 200 (Hair et al., 2010).

Considering the results of the EFA, items 4 and 8 were removed from the analysis since they did not contribute to the factor where they theoretically belong. Thus, the rotated factorial matrix was established for the instrument with 30 items (see **Table 3**).

**Table 3:** Items, communality and exploratory factor analysis (EFA) loadings Spanish version of MAIA.

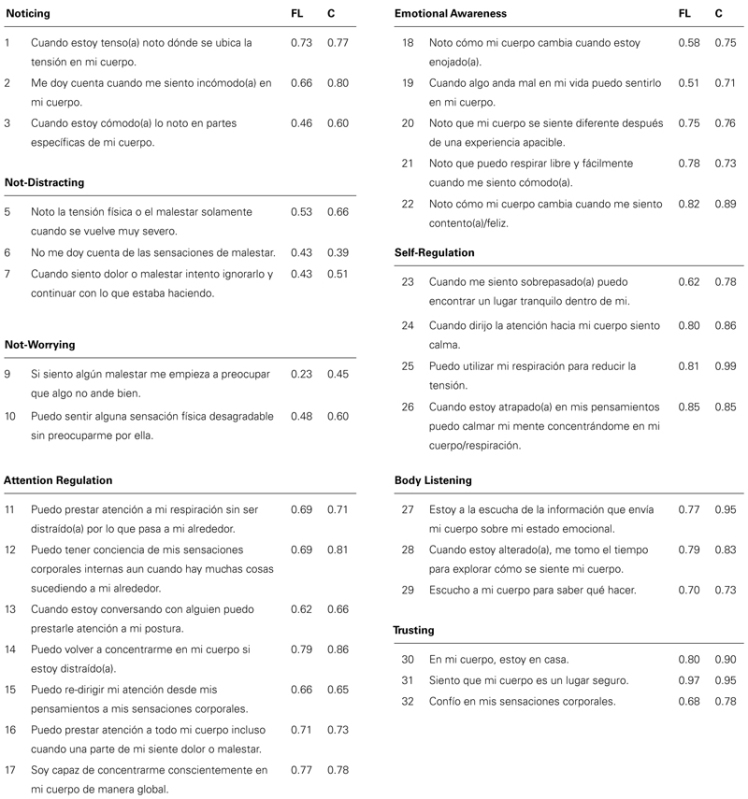

*Method for factor extraction: ML, Maximum Likelihood; Method the rotation: Normalized Promax. FL, Factor loading; C, Communality.*

Similar to the original scale, a structure of eight factors produced the best fit, however, item 9 achieved a factor loading of 0.23, considered insignificant for the sample size (Hair et al., 2010). Higher factor loadings for item 9 were achieved when the sample size increased: it was thus maintained to preserve the Not-Worrying subscale. The factor loadings of the other scale items varied from.43 to.97, which were considered satisfactory. The goodness of fit statistics of the model were χ^2^_(223)_ = 327.337 (*p* < 0.001), a CFI = 0.96, a GFI = 0.99, a TLI = 0.93 and a RMSEA = 0.046.

The observed χ^2^ value leads us to reject the hypothesis of an exact fit of the model. Considering that this statistic has the tendency to reject models when working with samples moderate to large in size (West et al., 2012), and the satisfactory values for the other indices, the results indicate a good fit for the eight factors model.

The reliability of the total scale based on the 30 items is 0.90 (Cronbach’s alpha based on standardized elements = 0.91, with reliability coefficients that vary between 0.40 and 0.86).

The subscale–subscale correlations analysis indicates higher associations between Self Regulation and Body Listening (*r* = 0.681, *p* < 0.01) and between Attention Regulation and Body Listening (*r* = 0.654, *p* < 0.01). The Not-Distracting subscale correlates poorly with Trusting (*r* = 0.189, *p* < 0.01) and demonstrates an inverse correlation with Not-Worrying (*r* = -0.133, *p* < 0.05). Not-Worrying does not show significant correlations with the other subscales. The item-scale correlations belonging to the subscales Not-Worrying and Not-Distracting have scores between -0.078 and 0.107. This indicates that these items do not differentiate people with high and low scores on the total scale. Correlations and reliability values for each subscale are presented in **Table 4**.

**Table 4:** Pearson product-moment correlations among the eight MAIA scales and Cronbach’s alpha.

	1	2	3	4	5	6	7	8
1. Noticing	0.637							
2. Not-Distracting	0.040	0.487						
3. Not-Worrying	-0.010	-0.133*	0.402					
4. Attention Regulation	0.474**	0.090	0.018	0.861				
5. Emotional Awareness	0.480**	-0.056	-0.096	0.436**	0.817			
6. Self-Regulation	0.285**	0.099	0.010	0.576	0.409	0.851		
7. Body Listening	0.399**	0.113	-0.031	0.654**	0.437**	0.681**	0.832	
8. Trusting	0.294**	0.189**	-0.021	0.519**	0.330**	0.577**	0.547**	0.855

Cronbach’s alpha on the diagonal.

*p < 0.05, **p < 0.01 (bilateral).

The second step of the analysis consisted in using the validation sample to perform CFA of the eight factors model. Through the CFA four models were built. Model 1 had eight correlated factors with factors loadings in the range of 0.35 and 0.96. Not all factors were significant. Model 2 included the covariance between the errors of the items 1–2, 1–3, 12–15, 13–14, 18–19, and 25–26. Item 6 was removed due to its low factorial load. This model yielded factor loadings between 0.40 and 0.97, all statistically significant (*p* < 0.001). This model had better indicators of goodness of fit than model 1. Model 3 preserved the covariance that had been incorporated in model 2 but eliminated the covariance between the factors Not-Worrying and Trusting; Not-Worrying and Attention Regulation; and Not-Worrying and Emotional Awareness. The indicators of goodness of fit did not significantly improve in this model. Finally, Model 4 removed the Not-Distracting factor: after removal of the covariance between this factor and other factors of the scale, the items of Not-Distracting resulted insignificant. The indicators of goodness of fit did not improve in this model. Goodness of fit statistics of the four models are presented in **Table 5**.

**Table 5:** Confirmatory factor analyses model fit indices.

	S-B χ^2^	*df*	*p*	CFI	TLI	RMSEA (IC 90%)	SRMR
Model 1	754.545	377	0.0001	0.894	0.878	0.063 (0.057–0.070)	0.060
Model 2	659.778	371	0.0001	0.919	0.905	0.056 (0.049–0.063)	0.059
Model 3	658.721	375	0.0001	0.908	0.905	0.056 (0.054–0.065)	0.059
Model 4	744.200	347	0.0001	0.919	0.908	0.060 (0.053–0.068)	0.072

*This fit-index was estimated with AMOS-18.*

**FIGURE 1:**
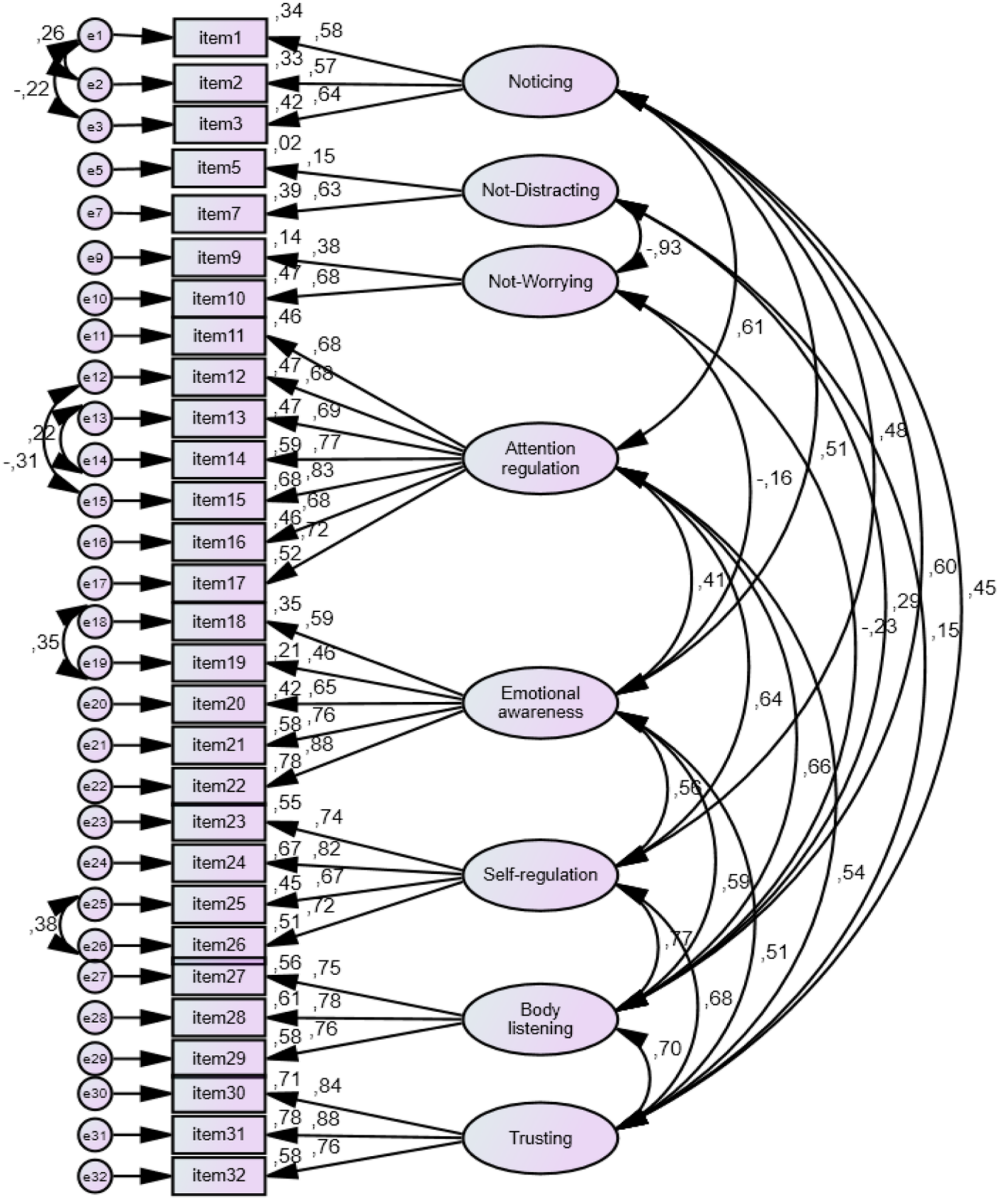
**Structural model of adaptation to Spanish of multidimensional assessment of interoceptive awareness (MAIA).** Path diagram model 2, standardized estimates.

The various models achieved through CFA present goodness of fit statistics similar to those of the original scale (Mehling et al., 2012). Model 2 presents significant factor loadings for the eight factors and the best goodness of fit statistics, therefore it was accepted as the model that best fits the data (see **Figure 1**).

## DISCUSSION

The aim of this study was to translate and adapt the MAIA into the Sapanish language, and to assess its psychometric properties in a Spanish speaking population. The Spanish tool was tested in a sample of 470 participants aged between 18 and 70 years, from the provinces of Valparaíso and Concepción, Chile. The adaptation was developed using a forward–backward translation, preserving the extension, format and the dimensional structure of the original scale. The cognitive interviews indicate comprehensiveness in most items. We identified difficulties in comprehension for items 5, 6, 7, and 15. Item 5 was reworded in order to avoid negation. Item 7 was reworded in order to better match the original meaning.

The EFA favored a model with a factorial structure of eight dimensions with low factorial loading for items 8 and 9. Items 4 and 8 were removed because they did not contribute to the factors that they theoretically belong to. A new rotated factorial matrix was established for the 30-item scale. This matrix showed a factorial structure of eight dimensions, similar to the original instrument, but with minor loading for item 9. We observed higher factorial loadings for item 9 as the sample size increased, we therefore decided to keep this item to preserve the dimension Not-Worrying.

The low contribution of item 4 (*Noto cambios en mi respiración tales como cuando se hace más lenta o más rápida, [I notice changes in my breathing. such as whether it slows down or speeds up.]*) to the subscale Noticing might be due to the order of this item following three items that refer explicitly to the experience of noticing a particular sensation in the body. The content of item 4 could be interpreted as relating to a “function” of the body rather than to the body itself.

The low contribution of item 8 (*Cuando siento dolor físico me enojo, [When I feel physical pain. I become upset]*) to the subscale Not-Worrying might be due to a translation issue. The word “upset” was translated as “enojo”: this word refers to the emotion anger and not to worry, which is what the subscale assesses. It is challenging to convey the meaning of ‘upset’ as used in English in the Spanish language.

Five of the eight scales showed alphas above 0.8, which indicate a good internal consistency. The reliability of the subscales Noticing (0.637), Not-Distracting (0.487) and Not-Worrying (0.402) is questionable. The reliability of the subscale Noticing was lower than found by Mehling et al., 2012 (0.69), Mehling et al., 2013 (0.74) and Bornemann et al., 2014 (0.76). Removing item 4 and reducing the subscale Noticing to three items might have weakened its reliability. Reliability usually increases when the number of items in the scale is increased (Cohen and Swerdlik, 2006). Low reliability of the subscale Not-Distracting might be due to: (a) the formulation using negation of item 6; (b) the small number of items in this subscale; and (c) the underlying construct. According to Mehling et al. (2012), the Not-Distracting subscale assesses the tendency not to use distraction to cope with discomfort, or not to ignore nor power through unpleasant sensations: these, in theory, are related to higher body awareness. In other words, this subscale assesses the ability to acknowledge, observe and attend unpleasant sensations. However, formulating this construct in its reverse form might lead to confusion; *under certain circumstances I prefer to ignore a given sensation* does not necessarily mean *I am unable to acknowledge it and to be aware of it*. It is unclear if “*Cuando siento dolor o malestar intento ignorarlo y continuar con lo que estaba haciendo*” *[When I feel pain or discomfort, I try to ignore it and to carry on with what I was doing]* means *I do not acknowledge the sensation*. It might simply mean that *even if I acknowledge it, it doesn’t paralyze me and I can carry on with what I was doing*. This subscale also presented low consistency in the English (0.66 in Mehling et al., 2012; 0.48 in Mehling et al., 2013) and German versions (0.56 in Bornemann et al., 2014), which might indicate that the underlying construct needs revision. The other possibility is that this subscale appears clearer to a particular sub-group, such as experts of body–mind practices. The results of the studies mentioned prior also support this possibility.

The low number of items (two), one with very low loading, likely explains the low consistency observed in the Not-Worrying subscale. Others have also observed low consistency here (0.67 in Mehling et al., 2012; 0.58 in Mehling et al., 2013; and 0.65 in Bornemann et al., 2014).

Subscale–subscale correlations analysis showed moderately high correlations between Body Listening and all the other subscales except Not-Worrying and Not-Distracting. These two subscales showed no correlation or low correlation with all other subscales. Mehling et al. (2013) report similar findings, indicating high association between Body Listening and Self-Regulation, and Body Listening and Emotional Awareness, as well as a lack of correlation of Not-Distracting and Not-Worrying with the other subscales.

The CFA supported a factorial structure of eight dimensions with goodness of fit statistics similar to the original scale.

One limitation of the present study is the small sample size and the relative lack of its representativeness biased by sex and educational level. The small sample size affects the conformation of the factorial structure and hinders the goodness of fit of the model, which has factorial loadings close to 0.40, at the limit for samples of around 200 participants (Hair et al., 2010; Lloret-Segura et al., 2014). This effect is particularly noticeable in item 9, where the load factor improved when larger samples sizes were modeled in the EFA.

While the translation and adaptation of the instrument was conducted considering various international guidelines, such as those outlined by Muñiz et al. (2013), it is suggested to continue the linguistic adjustment, particularly for items 6 and 8, which mainly affect the Not-Distracting and the Not-Worrying subscales: the reverse structure of these might pose added difficulty for respondents (Hartley, 2013). These factors have less than the minimum three items and thus threaten the dimension assessment (Long, 1983; Bollen, 1989; Abad et al., 2011), affecting the reliability of the subscales. In several solutions, these items did not reach sufficient statistical significance to load for a factor. This resulted in overlapping of the dimensions in some solutions, while grouping others into a single factor, hence, not discriminating satisfactorily.

We recommend reconsidering the order of the items in the scale. In the MAIA items are grouped by subscales and not randomly distributed as in Likert scales. Ordering items randomly prevents the establishment of a pattern of automatic responses. This also applies for reverse items, which were grouped within two subscales (Not-Distracting and Not-worrying), rather than being randomly distributed.

Regarding the questionable behavior of the Not-Distracting and Not-Worrying subscales, and that difficulties in these scales have been reported in other studies, we suggest revising these items. One solution could be to include items that better assess the construct measured by these factors, for example reverse items in their affirmative forms. Further, adding items in these subscales to include a minimum of four per factor might aid to promote the internal consistency of the scale, and its reliability.

Future development of the MAIA in the Chilean population should explore and provide evidence for convergent and divergent validity. Further studies with clinical and non-clinical populations, or samples with specific characteristics, are required to explore the differential performance of the items. Finally, applying item response theory (IRT) to model individual subject responses for a given ability may further enhance research efforts in this population. These developments would facilitate the future use of the scale in a professional context, beyond the research setting.

## CONCLUSION

The Spanish version of the MAIA showed satisfactory psychometric properties. An eight-factor model was built from the EFA, similar to the original scale. CFA confirmed the eight correlated factors model (Model 2). This model shows satisfactory goodness of fit statistics: χ^2^_(371)_ = 659.78, *p* = 0.0001; CFI = 0.92, TLI = 0.91 and RMSEA = 0.056 (IC 90%: 0.049–0.063). Altogether, these indices support the adequacy of the eight-factor model, with a goodness of fit similar to the original scale.

Regarding the subscales Not-Worrying and Not-Distracting, the low value of their reliability coefficients, the covariance between the errors for some items and the low factor loadings suggest a revision of these is warranted.

To conclude, the present study shows that the Spanish adaptation of MAIA is an appropriate tool to assess interoceptive awareness in the Chilean population. This favors the use of this tool for research purposes, and provides the possibility to study variables associated with psychological well-being, physical well-being and other interventions in the field of human health.

## Conflict of Interest Statement

The authors declare that the research was conducted in the absence of any commercial or financial relationships that could be construed as a potential conflict of interest.
